# Electrophysiological Advances on Multiple Object Processing in Aging

**DOI:** 10.3389/fnagi.2016.00046

**Published:** 2016-03-02

**Authors:** Veronica Mazza, Debora Brignani

**Affiliations:** ^1^Center for Mind/Brain Sciences (CIMeC), University of TrentoRovereto, Italy; ^2^IRCSS Centro San Giovanni di Dio, FatebenefratelliBrescia, Italy

**Keywords:** aging, multiple object processing, attention, working memory, EEG, enumeration, N2pc, contralateral delayed activity (CDA)

## Abstract

EEG research conducted in the past 5 years on multiple object processing has begun to define how the aging brain tracks the numerosity of the objects presented in the visual field for different goals. We review the recent EEG findings in healthy older individuals (age range: 65–75 years approximately) on perceptual, attentional and memory mechanisms-reflected in the N1, N2pc and contralateral delayed activity (CDA) components of the EEG, respectively-during the execution of a variety of cognitive tasks requiring simultaneous processing of multiple elements. The findings point to multiple loci of neural changes in multi-object analysis, and suggest the involvement of early perceptual mechanisms, attentive individuation and working memory (WM) operations in the neural and cognitive modification due to aging. However, the findings do not simply reflect early impairments with a cascade effect over subsequent stages of stimulus processing, but in fact highlight interesting dissociations between the effects occurring at the various stages of stimulus processing. Finally, the results on older adults indicate the occurrence of neural overactivation in association to good levels of performance in easy perceptual contexts, thus providing some hints on the existence of compensatory phenomena that are associated with the functioning of early perceptual mechanisms.

## Efficient Processing of Multiple Visual Objects in Young individuals

Processing multiple relevant objects concurrently allows for a coherent perception of the world. For this reason, multiple object analysis has been the focus of intensive research in several areas of cognitive neuroscience, ranging from attention to working memory (WM; Cavanagh and Alvarez, 2005; Baddeley, 2012).

Several cognitive models propose a distinction between at least two separate classes of mechanisms involved in multiple object analysis (Pylyshyn, 2001; Xu and Chun, 2009). Early individuation mechanisms provide a coarse representation of up to 3–4 objects simultaneously, allowing the visual system to individuate each object as being separate from others (Trick and Pylyshyn, 1993, 1994). Whereas the earlier proposals argued that such mechanisms operate in the absence of attention (Trick and Pylyshyn, 1993), recent research has suggested that simultaneous indexing of relevant items is tightly related to attention, being indeed one of its key functions (Cavanagh, 2011). Subsequent mechanisms, likely relying on the operation of visual WM, encode the individuated objects in greater details, ultimately leading to full identification. The proposed distinction between attentional individuation and WM mechanisms in multiple object processing has been supported empirically by behavioral and (to some extent) neuroimaging data (for a review, see Xu and Chun, 2009).

**Figure 1:**
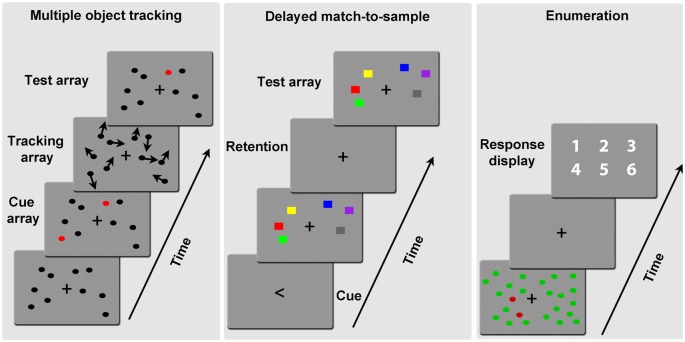
**Examples of the paradigms used to explore multiple object processing**. Left: in multiple object tracking tasks participants are typically presented with a varying number of elements and a cue indicating the targets (here the red circles; see cue array), before all elements start moving for a variable amount of time (tracking array). The movement trajectory (here indicated by the arrows) was linear and with a constant speed of 1°/s in Störmer et al. (2013a). A test array prompts participants to report if the probe element (the red circle in the example) was a target element or not. Center: in delayed match-to-sample judgments a set of elements is identified as the target by a cue (the arrow in the example), and participants need to retain the set of target elements for a certain amount of time (retention period, typically 1 s see Sander et al., 2011) for subsequent old/new recognition on a test array. Right: in enumeration tasks, a varying number of targets (here the red elements, as indicated at the beginning of the experimental session) is presented among distracters; participants report the number of targets either immediately or after a short delay (200 ms in Pagano et al., 2015, see example).

On the basis of behavioral and electrophysiological/event-related potential (EEG/ERP) results on multiple object analysis in enumeration tasks, Mazza and Caramazza (2015) have recently proposed a more detailed framework for the interpretation of the various stages involved in multiple object processing. The framework considers three mechanisms (reflected in three components of the EEG signal–N1, N2pc and contralateral delayed activity, CDA) that track the numerosity of the objects for different objectives. An early perceptual mechanism (100 ms post-stimulus onset, reflected in the N1) operates over the entire stimulus configuration, and allows for a coarse representation of the number of elements, irrespective of task relevance. A mid-latency attentive mechanism (200 ms post-stimulus, reflected in the N2pc) distinguishes the relevant elements from distracters, and additionally individuates up to 3–4 relevant elements. The set of individuated objects are subsequently maintained in a WM buffer (300 ms post-stimulus, reflected in the CDA) during quantity-to-symbol mapping. The similarity between the EEG patterns found in several tasks requiring multiple object processing—such as enumeration (Mazza and Caramazza, 2011; Ester et al., 2012), multiple object tracking (Drew and Vogel, 2008) and delayed match-to-sample (Vogel and Machizawa, 2004)—invites the inference that these mechanisms are implemented similarly during the execution of various tasks involving multiple target processing. In multiple object tracking tasks observers are required to keep track of a varying number of moving objects for subsequent recognition. In delayed match-to-sample paradigms, participants need to retain a set of elements for subsequent old/new recognition test. Finally, in enumeration tasks observers report the exact numerosity of a set of target elements presented simultaneously. More details about these tasks are provided in Figure 1.

Most of the EEG evidence gathered thus far on multiple object analysis comes from studies on young adults (18–30 years). However, the framework described above could also offer the basis for the characterization of multiple object processing throughout lifespan. Since the ability to process multiple objects simultaneously is required by numerous tasks, evaluating how it is modified by aging could provide hints on the integrity of the cognitive functions in older individuals. We review the most recent EEG studies on aging and multiple object processing by discussing the results within the framework outlined by Mazza and Caramazza (2015).

## Processing of Multiple Visual Objects in Old individuals: Is It Still Efficient?

Aging is typically equated with decline. Several findings support the equation by indicating a general age-related decline in perception, attention and WM relative to single object presentations (for a review, Verhaeghen, 2013). Accordingly, the impairment should be even more consistent when multiple rather than single targets are involved. In fact, the presence of age-related impairments in multiple target processing is controversial (see Zanto and Gazzaley, 2014). For instance, there is evidence of equivalent performance level in young and old adults for tasks where two inputs are processed simultaneously (e.g., Somberg and Salthouse, 1982; Hahn and Kramer, 1995), with a decline in performance visible only for larger sets of objects (Geary and Lin, 1998; Trick et al., 2005).

Until recently, it has remained an unresolved issue whether the age-related behavioral impairment in multiple target processing could be interpreted in terms of a decline equally affecting all the mental operations associated with the analysis of multiple objects, or whether a specific component is mainly involved. For instance, if the age-related impairments previously found in multiple object analysis were the consequence of a general slowing due to aging, all the neural and cognitive mechanisms involved in multiple object analysis should be delayed and/or suppressed (Salthouse, 1996). On the contrary, a delay or suppression of one specific mechanism would speak to a selective impairment in aging. The existing gap is partially explained by the extensive use of behavioral measures, which represent the final outcome of several stages of processing and thus are not ideal for distinguishing the contribution of a specific processing stage in stimulus analysis. Recent advances in neuroimaging techniques (and EEG, in particular) in terms of portability and noise reduction in signal extraction have allowed researchers to address this issue successfully.

In this article, we discuss recent attempts at investigating the age-related EEG/ERP correlates of the various stages of processing involved in in multiple object tracking, delayed match-to-sample and enumeration tasks (Figure 1).

### Early Perceptual Modulation of Multiple Objects: N1 (100–200 ms)

Seminal as well as more recent work on aging and multiple object tracking has shown a decline in performance for older adults (Trick et al., 2005; Störmer et al., 2011). Focusing on early posterior modulations, a recent ERP study on aging Störmer et al. (2013a) has indicated an N1 modulation that is associated to a behavioral impairment during multiple object tracking. Specifically, there was an N1 reduction for older adults (together with a delayed P1, a response considered to reflect sensory processing). This result is in line with other findings related to WM tasks, such as delayed match-to-sample judgments (Figure 1). In these tasks, older adults perform worse than the younger counterparts (e.g., Oscar-Berman and Bonner, 1985). ERP studies with sequential (Gazzaley et al., 2008) and simultaneous (Zanto et al., 2010) targets in these tasks have individuated the N1 as the first locus of neural modulation in aging. Overall, these studies suggest an early locus of age-related changes in multi-object analysis, at a stage where a relatively coarse representation of the overall number of elements in the visual field is formed. More precisely, the decrease in the N1 amplitude is considered as evidence of underactivation of the neural structure underlying early perceptual processing, and thus as a sign of age-related decline in perceptual processing.

Interestingly, recent studies have also shown the opposite modulation of N1 in aging. For instance, in some memory tasks with lateralized targets (namely, targets presented on the left or right side of the screen) the amplitude of the contralateral N1 is enhanced rather than reduced for older adults (Störmer et al., 2013b; for similar results also see Wiegand et al., 2014). One way to interpret this enhancement refers to the idea that aging involves compensatory re-organization of the visual functions, and that such compensatory phenomena may not be limited to late high-level processing but can also occur at early perceptual processes (see Talsma et al., 2006; De Sanctis et al., 2008).

### Attention Selection of Multiple Objects: N2pc (200–300 ms)

**Figure 2:**
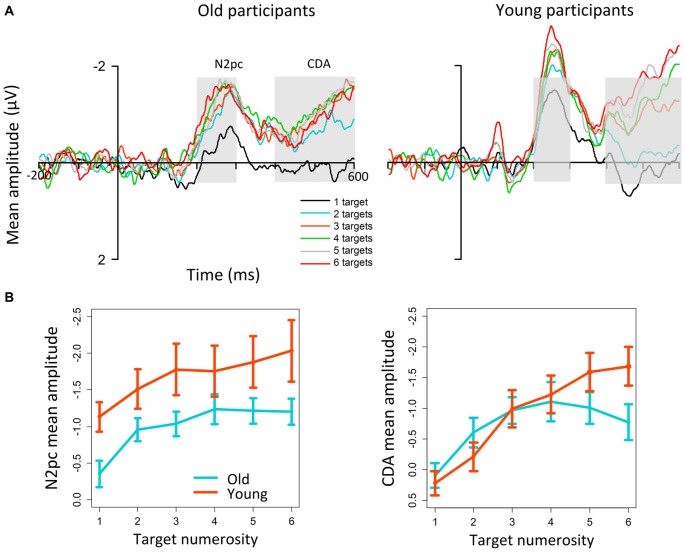
**N2pc and contralateral delayed activity (CDA) results on enumeration in aging (figure modified from Pagano et al., 2015)**. **(A)** Grand-average ERP waveforms for old and young participants as a function of target numerosity. A reduction of both N2pc (see gray area) and (partially) of CDA is visible for older adults. **(B)** The analysis on mean amplitude values for N2pc (180–300 ms) and CDA (400–600 ms) highlights that the age-related difference in N2pc amplitude is present for all target numerosities; in contrast, the CDA is equivalent in the two groups for up to three targets, and differentiates young and older participants only from approximately four elements.

In the delayed match-to-sample task mentioned above, Störmer et al. (2013b) found that the age-related increase in N1 was followed by a substantial reduction of the N2pc, an attentional response that reflects selective individuation of single (Luck and Hillyard, 1994) and multiple target objects (e.g., Ester et al., 2012; Pagano and Mazza, 2012; for a review, see Mazza and Caramazza, 2015). Thus, this result suggests that attentive selection of multiple relevant objects is impaired by age. Converging evidence comes from enumeration tasks in the elderly (Figure 1). Behavioral studies on aging (Watson et al., 2002, 2007) have found a decline in the enumeration speed of older adults, particularly when targets are presented in cluttered scenes (namely, together with distracters). Accordingly, Pagano et al. (2015) have recently found that older participants are progressively less accurate in enumerating target numerosities, and that this decrease is mirrored in an overall reduction of the N2pc relative to younger controls (Figure 2).

The N2pc reduction by age indicates that the attention-based mechanism reflected by this EEG response is overall less efficient in aging for the entire numerosity range (1–6 elements), and support previous interpretations (Watson et al., 2002) that attentional resources during enumeration are reduced by age. More in general, the extant findings of compromised selection of multiple targets are additionally consistent with previous work on single target search (Lorenzo-López et al., 2008) indicating a reduction of the N2pc amplitude in association with impaired target search in aging.

### Working Memory and Multiple Objects: CDA (300–600 ms)

The studies on WM in old participants have additionally disclosed a reduction of amplitude in the CDA (Vogel and Machizawa, 2004), a response reflecting the efficient maintenance of a limited set (3–4 targets) of selected elements in memory. For instance, Sander et al. (2011) highlighted a marked reduction of CDA in tasks where older participants had to encode and maintain a varying number of targets briefly presented in the visual field. The CDA reduction by age is more pronounced for older individuals with low accuracy (Wiegand et al., 2014). A recent study Jost et al. (2011) indicates an age-related variation only in the early phase of CDA, suggesting that the age-related impairment in WM is localized at an early stage of information retention, and that it is likely related to an inability to prevent the irrelevant information from accessing WM. Interestingly, CDA modulations are also able to track age-related differences when the items to be remembered are cued after stimulus offset (Duarte et al., 2013).

WM processes are also involved in the execution of some visual search tasks (Mazza et al., 2007; Wiegand et al., 2013), as well as in enumeration. Together with a reduction of N2pc, Pagano et al. (2015) found a subsequent reduction of CDA for older individuals when they enumerated targets among distracters (Figure 2). In contrast with the N2pc pattern and with previous studies on the CDA, the difference between older and younger individuals was visible only for the larger numerosities (>3 targets). This suggests that in enumeration tasks WM for small target numerosities (up to approximately 3–4 elements) is relatively well preserved in aging.

## Conclusions and Future Directions

EEG research on multiple object processing represents a new direction for evaluating the integrity of the cognitive functions in the elderly. Although not conclusive (for instance, the age of the older participants considered is quite homogeneous, ranging from 65–75 years; thus, it is currently unknown whether and how the processes and EEG components linked to multiple objects are additionally influenced by the age of the elderly), the few existing studies have started to delineate the temporal dynamics associated to neural and behavioral changes during the execution of various tasks requiring multiple object processing.

With respect to the question of whether aging influences one or multiple stages in multiple object analysis, the current findings point to multiple loci of neural changes in multi-object analysis: the same three EEG signals that track object numerosity in young adults are subject to changes in elderly. Specifically, the most consolidated result published thus far is a decrease of the EEG signal amplitudes for older adults (e.g., Sander et al., 2011; Wiegand et al., 2014), which is taken as evidence of age-related decline in perceptual mechanisms, attentive object individuation and WM operations.

Crucially, however, some studies did not find modulations at all stages of multiple object processing. This aspect argues against a pure account of aging in terms of overall processing slowing (e.g., Salthouse, 1996), which would predict that all the mechanisms involved in multiple object analysis become delayed and/or suppressed^1^. Additionally, it disproves the existence of “cascade effects”, according to which changes in one mechanism of multiple object analysis would simply propagate over all the subsequent mechanisms (as reflected by successive EEG/ERP responses). Indeed, a reduction of N2pc for the entire set of small target numerosity is sometimes followed by a reduction of CDA only for the largest numerosities of the set (Pagano et al., 2015). This pattern suggests a dissociation between an age-related decrement and/or slowing in processing efficiency that applies to all object quantities (reflected in the N2pc), and a selective age impairment in capacity limit, namely in the number of elements that can be processed simultaneously (reflected in the CDA). While the factors driving these dissociations should be examined more systematically, the differential involvement of perceptual mechanisms, attention and WM required for the execution of the tasks considered could explain the differences found across the paradigms examined here. For instance, differences in the involvement of WM for delayed match-to-sample vs. enumeration tasks could explain the discrepancy between some results present in the literature. Indeed, the extant studies indicating an age-related change in CDA (e.g., Sander et al., 2011) used delayed match-to-sample tasks, which likely rely on the WM system more heavily than the enumeration task used in the Pagano et al. (2015) study^2^.

A further interesting aspect emerging from the extant findings is that not all the EEG events related to aging represent a decrease in neural and cognitive activity. For instance, some of the EEG findings on the N1 reviewed here (Störmer et al., 2013b; Wiegand et al., 2014) suggest the existence of compensatory phenomena, specifically associated to early perceptual mechanisms. Previous fMRI studies have indicated the presence of neural hyper-activation in older adults associated to good level of performance compared to younger controls (for a review, Grady, 2012). This in turn has led to the formulation of theories on neural compensation (such as CRUNCH, see Reuter-Lorenz and Cappell, 2008), according to which neural hyper-activation has compensatory effects when the perceptual load imposed by the task is low, leading instead to neural hypo-activation and behavioral impairment in the case of high perceptual load. While it is currently unclear what factors cause hyper- vs. hypo-activation of N1, N1 increases have been observed when the amount of perceptual information is overall small (e.g., when only a few elements are presented, as in Störmer et al., 2013b). Thus, as proposed by some theories on compensation (Reuter-Lorenz and Cappell, 2008), perceptual load could predict the modulation of N1 in aging, and in turn show the existence of compensatory phenomena at early perceptual mechanisms. Future studies will explore more systematically whether N1, N2pc and CDA could represent mechanisms responsible for compensation in aging.

The investigation of the EEG correlates of multiple object processing may provide useful indication also in the evaluation of pathological aging, particularly in the diseases with an onset characterized by memory and attentional impairment, such as Alzheimer’s disease (AD). As AD is part of a clinical and biological continuum, it is of paramount importance to identify its preclinical phases, in order to intervene early in the trajectory of the disease to slow down or even halt its progression. For this reason, it is crucial to identify reliable markers of AD, able to capture early onset of the pathological aging and to distinguish between different etiologies (Albert et al., 2011; McKhann et al., 2011). Several EEG measures have been proposed as diagnostic markers for AD (e.g., Chapman et al., 2007), but the ones described in the article could be particularly suited to this aim. The pathogenesis of AD may begin dozens of years before the first cognitive symptoms appear (Hardy and Selkoe, 2002), affecting also the cortical areas involved in attention functions (Finke et al., 2013). Consistently, behavioral evidence shows that WM and attentive deficits occur not only at an early stage of the disease, but also in patients with mild cognitive impairment (MCI), a clinical syndrome commonly considered a prodromal of AD (Maylor et al., 2005, 2008; Neufang et al., 2011). In a delayed match-to-sample task, Newsome et al. (2013) provided the first evidence that CDA may be particularly sensitive to the earliest stages of the disease process. Participants were healthy elderly whose neuropsychological screening indicated risk for developing MCI. Consistently with a WM impairment, they displayed an abnormal CDA, with no significant differentiation between set sizes. A study by Cespón et al. (2013) on multiple domain- (but not single domain-) amnestic MCI patients showed a smaller N2pc in comparison to healthy participants during a Simon task, indicating the N2pc as a moderately good biomarker for distinguishing the two subtypes of MCI patients from healthy participants.

Finally, the EEG measures described here represent the ideal tool for investigating the temporal dynamics of the neural activity of the healthy aging brain, but they are limited in providing hints on the spatial sources of such activity. A good compromise for an optimal view on the time course of the interactions between neural networks is represented by MEG recordings (Rossini et al., 2007). However, to date studies on aging and evoked or induced neural activity with MEG are scanty, thus how the various neuronal assemblies in older individuals interact with one another during multiple object processing is largely unknown.

Overall, the picture emerging from the EEG correlates of multi-object analysis in the healthy older population is rather heterogeneous with respect to the findings in younger controls. This may be due to the limited number of studies currently published on this issue, but could also reflect an intrinsic feature of aging, namely a greater variability among individuals in the execution of various tasks as compared to early adulthood.

## Author Contributions

VM and DB contributed equally to the writing up of the mini review.

## Funding

The present work was funded by a Grant from the Italian Ministry of Health awarded to VM (Premio Giovani Ricercatori, grant number: 114/GR-2010-2314972). The funder had no role in study design, data collection and analysis, decision to publish, or preparation of the manuscript.

## Conflict of Interest Statement

The authors declare that the research was conducted in the absence of any commercial or financial relationships that could be construed as a potential conflict of interest.

## Footnotes

^1^Older individuals usually suffer from peripheral deficits, such as low visual acuity. This aspect could influence the entire sensory, perceptual and “cognitive” processing of stimuli. However, participants are typically included in the experimental sample only if they have normal or corrected-to-normal vision. They are also tested (either informally or more formally, e.g., see Sander et al., 2011) for some basic aspects of vision, such as visual acuity. In addition, since peripheral processing occurs at early stages of stimulus processing, the related deficits should affect all the subsequent EEG components (e.g., perceptual, attentive, mnestic). In contrast, the findings reported in the review indicate different effects of aging on the various mechanisms (as reflected by the different EEG components) considered here. Arguably, this aspect convincingly dismisses the possibility that the patterns found in these studies are exclusively driven by a loss in peripheral processing efficiency.

^2^Delayed match-to-sample tasks also measure other aspects of cognition, such as executive functions.
